# Supplementary material for: COVID-19 in breast cancer patients: a cohort at the Institut Curie hospitals in the Paris area
Source: Breast Cancer Res. 2020 May 28;22:55. doi: 10.1186/s13058-020-01293-8 (PMC7254663; doi:10.1186/s13058-020-01293-8)

**Supplementary Methods 1: Data collected in the registry**

| **Patient history and cancer**  **History** | Date of birth Primary Cancer Treatment setting  Hormone receptor status (ER, PR)  HER2 status*  Previous adjuvant radiotherapy (fields)  Date of primary tumor diagnosis  Date of first metastasis  Number of prior lines of treatment (if stage IV)  Metastatic sites  BMI  Comorbidities  Chronic treatments  Steroids, NSAIDS, ACE inhibitor/ARB, Anticoagulants,  Immunosuppressive drugs |
| --- | --- |
| **Symptoms and COVID-19 data** | Primary reason for registry  Site of COVID-19 diagnosis  Symptoms type  Date of first symptoms  At first diagnosis (and, if available > 30 days before):  Hemoglobin (g/dL)  Platelets counts (G/L)  Neutrophils counts (G/L)  Lymphocytes count G/L)  Lactate dehydrogenase (ULN)  CRP  PCT  Ferritin  Fibrinogen (g/L)  Prothrombin time (expressed in %)  SGOT (ULN)  SPGT (ULN)  Date of Chest CT-scan Date of RT PCR test and results  SARS-CoV-2 treatment, if initiated on the same day  Antibiotics  Corticosteroids  Choloroquin  Hydroxyclhoroquin  Other anti-viral  Other |
| **Radiological data** | Date of Chest CT-scan  Chest CT-scan results Central reviewed performed  Extent of lesions  Predominant type of lesions  Residual toxicity of Mammary radiotherapy |
| **Healthcare** | Related to oncological care  Treatment received in the last 30 days  Date of the last treatment  Related to COVID-19 infection  Hospitalization status  Site of Hospitalization  Date of hospitalization  Date of discharge  Type of follow up  Oncological treatment delay |
| **Follow up calls** | Date of follow up Time since the first screening  Follow up modality  Hospitalization (ICU?)  Outpatients  Other  Symptoms update  Reason to stop follow up  Death?  Date of death or last follow up |

ER: Estrogen Receptor; PR: Progesterone Receptor**;** BMI: body mass index; ULN: Upper Limit of Normal range; NSAID: non-steroidal anti-inflammatory drugs; ACE: angiotensin-converting enzyme; ARB: angiotensin II receptor blockers.

**Supplementary Methods 2**

Patient flow. Starting on March 9, 2020, ICH provided information about COVID-19 symptoms and guidelines to patients by means of regular e-mail and text messages. Follow-up consultations were postponed. Consultations not requiring physical examination or detailed explanations were replaced by teleconsultations. Patients were systematically subjected to COVID-19 symptom screening and body temperature checks at the hospital entrances. Those with COVID-19 symptoms were directed to specific emergency room areas and were tested. When necessary, patients with suspected or proven COVID-19 were hospitalized in specific wards or transferred to internal or external ICU. Of note, Paris and its area did not run out of standard inpatients or ICU beds.

Surgery. The French hospital emergency response plan (“*plan blanc”*) required postponement of all non-urgent surgery. ICH surgeons complied with the national guidelines for breast cancer patients issued by learned societies [1], included postponement of surgery for patients at high risk of severe COVID-19, in whom an alternate treatment option was available (eg, neoadjuvant endocrine therapy was proposed in elderly ER+ breast cancer patients). Patients were systematically tested for SARS-CoV-2 RNA (nasopharyngeal swabs) in the two days prior to general anesthesia, even in the absence of symptoms.

Medical oncology. Internal guidelines were applied at ICH starting from March 9, in line with national guidelines for breast cancer patients [1]: ongoing chemotherapy was not discontinued. Neoadjuvant chemotherapy was avoided in patients with smaller tumors (< 3 cm), even including triple-negative and HER2-positive subtypes. Adjuvant chemotherapy indications were mostly maintained, but situations associated with a marginal survival benefit had to be discussed with the patients. Dose-dense anthracycline regimens were discouraged. Granulocyte colony-stimulating factor prophylaxis was systematically used for anthracycline-based regimens and docetaxel, with the docetaxel dose reduced to 75 mg/m^2^. In patients experiencing COVID-19 symptoms (proven or unproven), the next chemotherapy cycle was delayed until 14 days after the first day of symptoms, pending the clearance of hyperthermia and dyspnea. Endocrine therapy indications and doses were not modified. Targeted therapy (including CDK4/6 and PARP inhibitors) indications and doses were not modified, except for alpelisib and everolimus. Initiation of a new line of therapy with alpelisib was discouraged, but ongoing treatment was maintained. Everolimus was discontinued in all patients, except for a handful of patients with prolonged objective response. Atezolizumab indications and doses were not modified, but close monitoring of these patients was recommended. When used as comedication for chemotherapy, corticosteroid doses were systematically reduced by one-half and were discontinued whenever possible.

Radiation therapy. Indications and ongoing treatments were not modified, but adjuvant radiotherapy was hypofractionated whenever possible [1, 2].

Palliative care. For patients with advanced metastatic breast cancer and/or severe pre-existing comorbidities, a dedicated COVID-19 videoconference multidisciplinary meeting was held daily to discuss the medical and ethical relevance of antiviral treatments and transfer to ICU. Palliative care for breast cancer patients during the COVID-19 pandemic was conducted according to the guidelines issued by the French society of palliative care [3].

1. Gligorov J, Bachelot T, Pierga JY, Antoine EC, Balleyguier C, Barranger E, Belkacemi Y, Bonnefoi H, Bidard FC, Ceugnart L *et al*: **[COVID-19 and people followed for breast cancer: French guidelines for clinical practice of Nice-St Paul de Vence, in collaboration with the College Nationale des Gynecologues et Obstetriciens Francais (CNGOF), the Societe d'Imagerie de la FEMme (SIFEM), the Societe Francaise de Chirurgie Oncologique (SFCO), the Societe Francaise de Senologie et Pathologie Mammaire (SFSPM) and the French Breast Cancer Intergroup-UNICANCER (UCBG)]**. *Bull Cancer* 2020.

2. Loap P, Kirova Y, Takanen S, Crehange G, Fourquet A: **Breast radiotherapy in the context of the COVID19 pandemic: practical tips in an epidemic period and advice for resuming activity at the end of the crisis**. *Cancer Radiother* 2020, **in press**.

3. [**http://www.sfap.org/actualite/outils-et-ressources-soins-palliatifs-et-covid-19**](http://www.sfap.org/actualite/outils-et-ressources-soins-palliatifs-et-covid-19)

**Supplementary Table 1**

Impact of systemic treatment modalities on COVID-19 severity (ICU admission or death), univariate analyses.

| Systemic treatment  received within 1 month before COVID-19 diagnosis | Odds ratio | 95%CI | P value |
| --- | --- | --- | --- |
| Chemotherapy | 1.6 | [0.3-7.7] | 0.6 |
| Endocrine therapy | 0.8 | [0.1-4.7] | 0.8 |
| Targeted therapy | 0.8 | [0.1-4.3] | 0.8 |

**Supplementary Figure 1: Radiation therapy and COVID-19 features**

Presence of pleural or lung metastases (inner circle), radiation therapy sequelae (semiquantitative estimates, green circle) and extent of COVID-19 lung disease (semiquantitative estimates, red circle) are displayed by irradiation fields (blue circle) for each of the 59 COVID-19 patients. Patients who died are surrounded in black. CT scans not available for central review are not displayed on the graph (marked as ‘not done’).

**
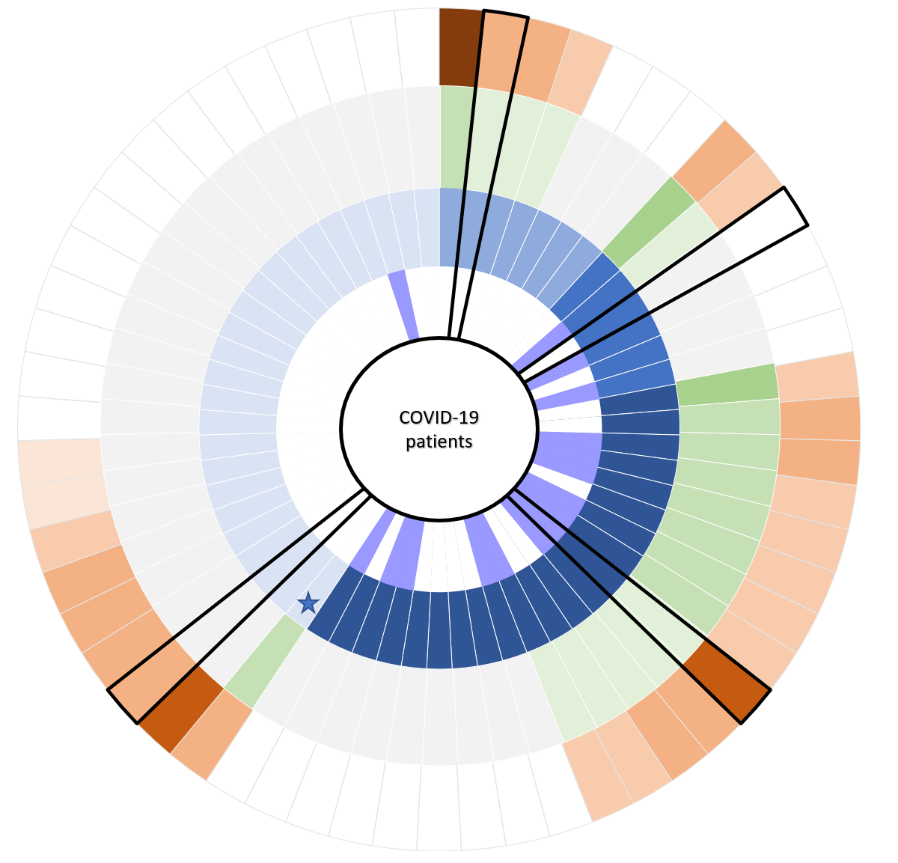
**


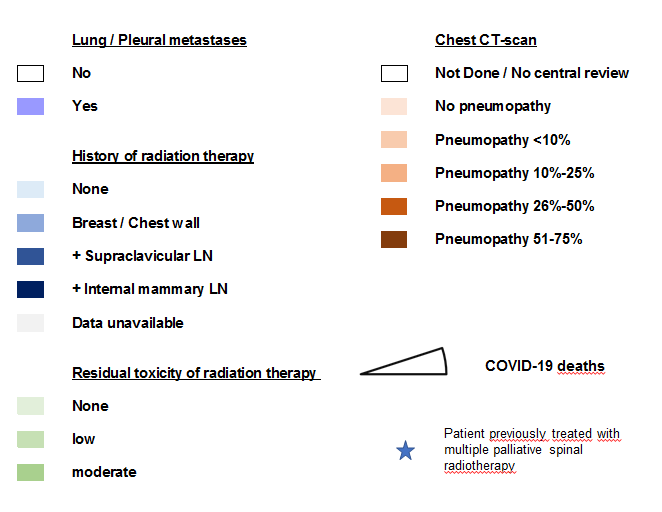

Supplement: Supplementary file 1 — Additional file 1. [file 13058_2020_1293_MOESM1_ESM.docx]
